# Osteogenic Potential of Pre-Osteoblastic Cells on a Chitosan-*graft*-Polycaprolactone Copolymer

**DOI:** 10.3390/ma11040490

**Published:** 2018-03-26

**Authors:** Anthie Georgopoulou, Maria Kaliva, Maria Vamvakaki, Maria Chatzinikolaidou

**Affiliations:** 1 Department of Materials Science and Technology, University of Crete, Voutes Campus, 70013 Heraklion, Greece; anthieg87@yahoo.com (A.G.); kalivm@iesl.forth.gr (M.K.); vamvakak@iesl.forth.gr (M.V.); 2 Institute of Electronic Structure and Laser, Foundation for Research and Technology-Hellas, Nikolaou Plastira 100, 70013 Heraklion, Greece

**Keywords:** biomaterial, chitosan, polycaprolactone, osteogenic potential, bone tissue engineering, pre-osteoblastic cells

## Abstract

A chitosan-*graft*-polycaprolactone (CS-*g*-PCL) copolymer synthesized via a multi-step process was evaluated as a potential biomaterial for the adhesion and growth of MC3T3-E1 pre-osteoblastic cells. A strong adhesion of the MC3T3-E1 cells with a characteristic spindle-shaped morphology was observed from the first days of cell culture onto the copolymer surfaces. The viability and proliferation of the cells on the CS-*g*-PCL surfaces, after 3 and 7 days in culture, were significantly higher compared to the cells cultured on the tissue culture treated polystyrene (TCPS) control. The osteogenic potential of the pre-osteoblastic cells cultured on CS-*g*-PCL surfaces was evaluated by determining various osteogenic differentiation markers and was compared to the TCPS control surface. Specifically, alkaline phosphatase activity levels show significantly higher values at both time points compared to TCPS, while secreted collagen into the extracellular matrix was found to be higher on day 7. Calcium biomineralization deposited into the matrix is significantly higher for the CS-*g*-PCL copolymer after 14 days in culture, while the levels of intracellular osteopontin were significantly higher on the CS-*g*-PCL surfaces compared to TCPS. The enhanced osteogenic response of the MC3T3-E1 pre-osteoblasts cultured on CS-*g*-PCL reveals that the copolymer underpins the cell functions towards bone tissue formation and is thus an attractive candidate for use in bone tissue engineering.

## 1. Introduction

Bone is a highly vascularized organ, providing shape, mechanical support and protection to vital organs and facilitating movement [1]. The functional restoration of bone defects resulting from trauma or bone diseases such as osteoporosis, arthritis and cancer is required for functional and aesthetic reasons [2]. The use of an appropriate material onto which osteoblastic cells attach, proliferate and differentiate is a major challenge in the replacement of the damaged tissue and the restoration of the impaired functionality [3]. During the last decades, a wide range of biomaterials have been employed as bone substrates, including bioactive ceramics, bioactive glasses, natural or chemical/synthetic polymers and their composites [4]. Biodegradable materials, both of natural or synthetic origin are particularly attractive for use in many tissue engineering applications, because a second surgery, after implantation, is avoided [5]. 

Chitosan (CS) is a natural, linear polysaccharide, derived from fully or partially deacetylated chitin [6]. A characteristic of chitosan is the presence of amino groups along the polymer chain which allows it to form complexes with anionic molecules such as lipids, DNA, proteins and negatively charged synthetic polymers [7]. Moreover, it is a non-toxic, non-immunogenic, biodegradable and biocompatible polymer with antibacterial and analgesic properties [8,9,10]. Therefore, chitosan constitutes an ideal material for wound dressing, drug delivery and tissue engineering applications such as skin and nerve regeneration [5,11]. On the other hand, its poor mechanical properties, plasticity and brittleness are the main limitations for its use in many applications [5]. Graft copolymerization [12,13] and blending with other polymers [14,15,16] have been employed as common approaches to overcome these limitations. 

Polycaprolactone (PCL) is a synthetic biodegradable and biocompatible polyester with good mechanical properties, high plasticity, non-toxicity and gradual resorption following implantation [5]. Despite the lack of bioactive functional groups and the hydrophobic character of PCL, several studies propose the use of PCL scaffolds to promote the proliferation of cells and extracellular matrix production [17,18,19]. PCL copolymers and blends with other natural or synthetic polymers have been frequently used as a biocompatible material both in soft and hard tissue engineering applications including cartilage and bone regeneration [18,19]. 

Previous studies have shown that CS/PCL blends are promising for use in skin and osteochondral tissue engineering, because they combine the biocompatibility and biological interactions of chitosan with the suitable mechanical properties of polycaprolactone [20]. Moreover, our group has shown that CS-*g*-PCL graft copolymers promote the viability and proliferation of Wharton’s jelly mesenchymal stromal cells (WJ-MSCs) and could be used in tissue regeneration [21]. In another study from our group we report on the immunomodulatory potential of CS-*g*-PCL compositions 50/50 and 78/22 wt %, showing that both indicate an anti-inflammatory activity that was higher by increasing the chitosan content from 50 to 78 wt %. 

The present study focuses on the evaluation of the in vitro biological response of MC3T3-E1 pre-osteoblastic cells cultured onto CS-*g*-PCL surfaces and their osteogenic potential in bone tissue engineering. First, the adhesion and morphology of the pre-osteoblastic cells on the CS-*g*-PCL surfaces were investigated using scanning electron microscopy (SEM) and confocal laser fluorescence microscopy. Moreover, the viability and proliferation of the cells at different time periods of culture were evaluated using the colorimetric assay PrestoBlue^®^ (ThermoFisher Scientific, Waltham, MA, USA) . Finally, the ability of the graft copolymer to promote the differentiation capacity of the pre-osteoblastic cells was assessed by determining specific early and late markers of osteogenesis, such as alkaline phosphatase activity, collagen production, calcium biomineralization and endogenous osteopontin expression. 

## 2. Materials and Methods 

### 2.1. Materials

A CS-*g*-PCL copolymer, with 78 wt % CS content, was used to coat 15 mm diameter glass coverslips. For the synthesis of the CS-*g*-PCL graft copolymer, low molecular weight chitosan (Sigma-Aldrich, St. Louis, MO, USA), with degree of deacetylation (DD) of ~85%, as determined by proton nuclear magnetic resonance (^1^H NMR) spectroscopy, was used. Poly(ε-caprolactone) bearing a carboxylic acid end-group (PCL-COOH), with a degree of polymerization of 45, as determined by ^1^H NMR analysis, was synthesized by ring opening polymerization using stannous octoate as the catalyst and glycolic acid as the initiator, and was chemically grafted along the chitosan backbone via the primary amino groups. Phosphate buffer saline (PBS), bovine serum albumin (BSA), Triton X-100, acetic acid, Direct red 80, Alizarin red S, cetylpyridinium chloride (CPC), *p*-nitrophenyl phosphate, Bradford reagent, paraformaldehyde, ascorbic acid, β-glycerolphosphate and dexamethasone, 4′,6-Diamidino-2-Phenylindole (DAPI) dihydrochloride, FITC-conjugated anti-vinculin antibody (F7053) and TRITC-phalloidin conjugated antibody (P1951) were purchased from Sigma-Aldrich (St. Louis, MO, USA). PrestoBlue^®^ reagent for cell viability and proliferation, minimum essential Eagle’s medium (α-MEM), L-glutamine, trypsin/EDTA, penicillin/streptomycin, fetal bovine serum (FBS), To-Pro^®^-3 iodide, actin from rabbit muscle were purchased from Molecular Probes by Life Technologies, ThermoFisher Scientific Waltham, MA, USA). 

### 2.2. Preparation of the CS-g-PCL Samples

CS-*g*-PCL surfaces on glass substrates were prepared by spin coating using a SPS spin coater (model Spin 150). 140 μL of a 1% *w*/*v* copolymer solution in H_2_O/CF_3_COOH 50% *v*/*v* was spun at 2000 rpm for 160 s. The coating was dried under high vacuum at 60 °C for 24 h before being neutralized by rinsing with 0.1 M NaOH solution for several minutes, washed with water and dried under a N_2_ flow.

### 2.3. Biomaterial Characterization

The synthesized graft copolymer was characterized by ^1^H NMR spectroscopy and Attenuated Total Reflection-Fourier transform infrared (ATR-FTIR) spectroscopy. ^1^H NMR spectra were obtained on a Bruker AMX-500 spectrometer (Billerica, MA, USA) by dissolving the samples in a CF_3_COOD:D_2_O (1:1) solvent mixture at 45 °C. The ATR-FTIR spectra were recorded on a Nicolet 6700 spectrometer (ThermoFisher Scientific, Waltham, MA, USA).

### 2.4. Cell Culture of Pre-Osteoblasts

MC3T3-E1 pre-osteoblastic cells were isolated from newborn mouse calvaria. Cells were obtained from DSMZ GmbH (DSMZ No: ACC 210 Braunschweig, Germany) and have the capacity to differentiate into osteoblasts and osteocytes in vitro. Pre-osteoblastic cells were cultured in α-ΜΕΜ medium supplemented with 10% fetal bovine serum (FBS), 2 mM L-glutamine and 1% penicillin/streptomycin. Once a week, when the cells reached confluence, they were passaged using trypsin/EDTA and were resuspended in culture medium. Next, 3 × 10^4^ and 5 × 10^4^ MC3T3-E1 cells were used for viability/proliferation and differentiation assays, respectively and were seeded onto the sterilized CS-*g*-PCL surfaces. Samples were placed in 24-well Corning^®^ plates, where tissue culture polystyrene were used as control substrates. All experiments were carried out using cells between passage 8 and 15.

### 2.5. Adhesion and Morphology of the Pre-Osteoblasts on the CS-g-PCL Surfaces 

The morphology of the pre-osteoblast cells on the CS-*g*-PCL surfaces was monitored by means of scanning electron microscopy (SEM) (JEOL JSM-6390 LV, Tokyo, Japan) after 3 days of incubation. At each time point, cells were rinsed twice with 1 M PBS buffer and were fixed with 2% *v*/*v* para-formaldehyde and 2% *v*/*v* glutaraldehyde for 15 min at room temperature and dehydrated in increasing concentrations (30% *v*/*v*–100% *v*/*v*) of ethanol. Samples were then dried in a critical point drier (Baltec CPD 030, Baltec, Los Angeles, CA, USA), sputter-coated with a 20 nm thick layer of gold (Baltec SCD 050, Baltec, Los Angeles, CA, USA) and observed under a microscope at an accelerating voltage of 15 kV.

Actin distribution and focal adhesion points of the cells were observed using confocal laser fluorescence microscopy. A suspension of 2 × 10^4^ cells were cultured on the polymer surface and after 3 days the medium was removed and the samples were washed twice with PBS. Cells were fixed with 4% para-formaldehyde for 15 min and permeabilized with 0.1% Triton X-100 in PBS. The non-specific binding sites were blocked with 2% BSA solution in PBS for 30 min. Samples were stained using fluorescein isothiocyanate-conjugated anti-vinculin antibody (1/100) (TRITC-phalloidin conjugate, Sigma-Aldrich, St. Louis, MO, USA) and tetramethyl rhodamine isothiocyanate-conjugated phalloidin (1/40) (Molecular Probes by Life Technologies, ThermoFisher Scientific, Waltham, MA, USA) for 50 and 30 min, respectively. For cell nuclei staining 100 μL of a DAPI dihydrochloride solution (1/100) were added on top for another 10 min. Samples were rinsed twice with PBS and were observed under a Leica DM IRBE laser scanning confocal microscope using a 40-fold magnification objective lens (Leica, Wetzlar, Germany). 

### 2.6. Viability and Proliferation of Pre-Osteoblasts on the CS-g-PCL Surfaces

3 × 10^4^ pre-osteoblastic cells were used for the viability and proliferation assay. The metabolic activity of the cells on the CS-*g*-PCL surfaces was assessed with the PrestoBlue^®^ assay. When cells are viable, the nontoxic metabolic indicator resazurin, is reduced to a red product resorufin, which can be detected photometrically. At each time point, namely after 1, 3 and 7 days in culture, 400 μL PrestoBlue^®^ reagent, diluted in primary culture medium (a-MEM) (1:10), was added directly to each well and was incubated at 37 °C for 30 min. The supernatants of the samples were transferred to another 24-well plate and the absorbance (570 nm and 600 nm) was measured using a spectrophotometer (Synergy HTX Multi-Mode Microplate Reader, BioTek, Winooski, VT, USA). Absorbance units were translated to cell number after using a calibration curve. Two independent experiments were performed in triplicates. 

### 2.7. Osteogenic Response of the Pre-Osteoblastic Cells on the CS-g-PCL Surfaces 

#### 2.7.1. Alkaline Phosphatase (ALP) Activity

An enzymatic activity assay was used to measure the levels of alkaline phosphatase activity expressed from the pre-osteoblastic cells cultured on the CS-*g*-PCL surfaces. Cells were cultured for 4, 7 and 14 days in osteogenic medium (primary medium supplemented with ascorbic acid, sodium glycerophosphate and dexamethasone (50 μg/mL, 0.1 μΜ, and 10 nM, respectively) and at each time point they were harvested by trypsin-EDTA and collected by centrifugation. Pellets were dissolved in 100 μL lysis buffer (0.1% Triton X-100 in 50 mM Tris-HCl pH 10.5) and were subjected to two freeze-thaw cycles from −80 °C to room temperature. Then, 100 μL of a 2 mg/mL *p*-nitrophenyl phosphate (pNPP, Sigma, St. Louis, MO, USA) substrate in 50 mM Tris-HCl at pH 10 with 2 mM MgCl_2_ were added to each sample and incubated at 37 °C for 60 min. The reaction was stopped with the addition of 50 μL 1 N NaOH. Absorbance was measured using a Synergy HTX plate reader (BioTek, Winooski, VT, USA) at 405 nm and was correlated to equivalent amounts of para-nitrophenol using a calibration curve. Alkaline phosphatase activity was normalized to cellular protein levels and was measured by the Bradford assay.

#### 2.7.2. Alizarin Red Staining

Alizarin red was used to stain the calcium deposits in the extracellular matrix of the pre-osteoblasts. Cells were cultured for 7 and 14 days in osteogenic medium (as described in Section 2.7.1) and at each time point of 7 and 14 days they were fixed with 4% paraformaldehyde for 15 min, rinsed twice with PBS and stained with 300 μL of 2% alizarin red S at pH 4.1 for 30 min. Then, cells were rinsed three times with H_2_O in order to remove the excess stain. Cetylpyridinium chloride (CPC) was used to quantify the accumulation of calcium deposits by dye extraction. 300 μL of 10% CPC in 10 mM sodium phosphate buffer solution (pH 7) were added to each sample and was incubated for 1 h under shaking. Absorbance was measured using a Synergy HTX plate reader at 550 nm. Absorbance measurements were normalized to the cell number, measured by the PrestoBlue^®^ assay prior to staining.

#### 2.7.3. Collagen Production in the ECM 

The Sirius Red Dye assay (Direct red 80, Sigma-Aldrich, St. Louis, MO, USA) was used to measure total collagen levels secreted from the pre-osteoblastic cells in the culture medium. Briefly, at each time point, 100 μL of culture medium was mixed with 1 mL 0.1% Sirius Red Dye and were incubated for 30 min at room temperature. After centrifugation of the samples at 15,000 *g* for 15 min, the pellets were washed with 0.1 N HCl in order to remove the non-bound dye. Samples were finally centrifuged at 15,000 *g* for 15 min and were dissolved in 500 μL 0.5 N NaOH. Absorbance was measured using a Synergy HTX plate reader at 530 nm. The absorbance measurements were correlated to the concentration of collagen type I using a calibration curve. 

#### 2.7.4. Endogenous Expression of Osteopontin Using in-Cell Enzyme-Linked Immunosorbent Assay (ELISA)

Osteopontin is a phosphorylated glycoprotein, which is involved in bone mineralization. In our study, the levels of endogenous osteopontin expressed from cells cultured on the CS-*g*-PCL surfaces and the tissue culture treated polystyrene (TCPS) control were measured using the in-cell enzyme-linked immunosorbent assay (ELISA). First, 5 × 10^4^ cells/well were cultured on the two materials surfaces for 10 days. At this time point, cells were fixed with 4% *v*/*v* paraformaldehyde for 15 min, before permeabilization with 0.1% *v*/*v* and blocking with 2% *w*/*v* BSA for 1 h at room temperature. Samples were incubated with anti-mouse primary antibody (1/1000) in 2% BSA/PBS for 2 h at 4 °C (300 μL) and after washing away the unbound antibody, an anti-rabbit IgG H&L antibody was added on top at 1/1000 dilution for 2 h (300 μL). Then, the enzyme substrate (TMB) (100 μL) is added and the reaction produces a color change signal, in proportion to the amount of the osteopontin levels. Finally, 100 μL of sulfuric acid stock solution was added, which changes the color from blue to yellow. The absorbance was measured using a Synergy HTX plate reader at 450 nm and valued by normalization to the cell number as percentage over the background. 

#### 2.7.5. Endogenous Expression of Osteopontin and Visualization by Means of Confocal Laser Fluorescence Scanning Microscopy

The endogenous expression of osteopontin (OPN) from cells cultured on the CS-*g*-PCL copolymer for 10 days was examined using a confocal laser scanning fluorescence microscope (CLSM, Leica DM IRBE, Wetzlar, Germany). At this time point, cells were fixed with 4% paraformaldehyde for 15 min, before permeabilization with 0.1% *v*/*v* Triton and blocking with 2% *w*/*v* BSA for 1 h at room temperature. Samples were incubated with anti-mouse primary antibody at a dilution of 1:1000 in 2% BSA/PBS for 2 h at 4 °C. A FITC-conjugated anti-rabbit IgG H&L was used as a secondary antibody at a 1:1000 dilution. For this experiment cells cultured on cover slips for 10 days were used as a control.

### 2.8. Statistical Analysis

Statistical analysis was performed using the student’s *t*-test (GraphPad Prism version 5 software, GraphPad, La Jolla, CA, USA) to evaluate the significance of the differences between the CS-*g*-PCL surfaces and the TCPS control surfaces. A *p* value of <0.05 was considered significant. 

## 3. Results

### 3.1. Synthesis and Characterization of the CS-g-PCL Copolymer

The CS-*g*-PCL copolymer was synthesized via a three-step procedure as described in our previous study [21]. Briefly, first CS is modified to an organophilic precursor, by the interaction with sodium dodecyl sulfate (SDS) to form an SDS/chitosan complex. Next, the activated carboxylic acid end-group of PCL, obtained from the reaction of PCL with *N,N*′-Dicyclohexylcarbodiimide (DCC) and *N*-Hydroxysuccinimide (NHS), is covalently grafted onto the amine moieties of CS by an amine coupling reaction. Finally, the CS-*g*-PCL copolymer is obtained by removing the SDS by precipitation in a Tris buffer solution. 

**Figure 1:**
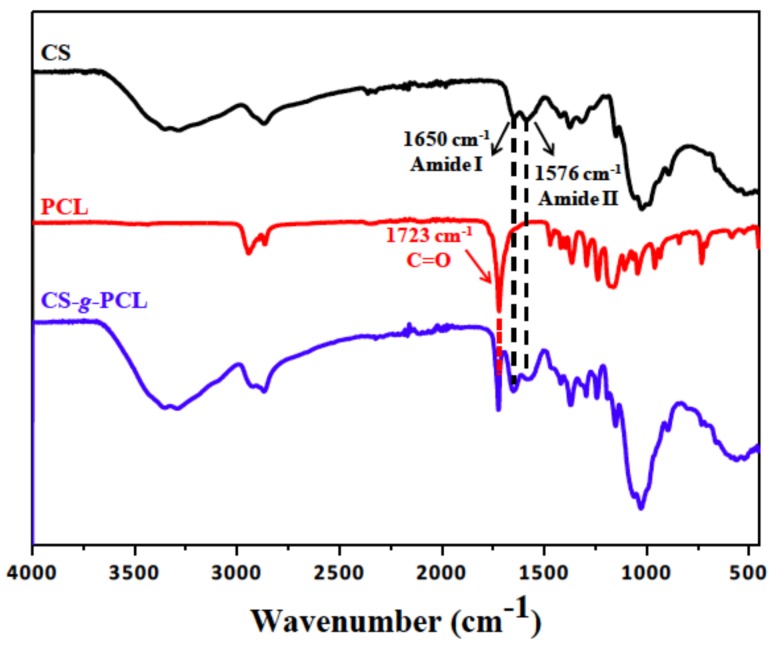
Attenuated Total Reflection-Fourier transform infrared (ATR-FTIR) spectrum of chitosan (CS), polycaprolactone (PCL) and the synthesized graft copolymer CS-*g*-PCL.

**Figure 2:**
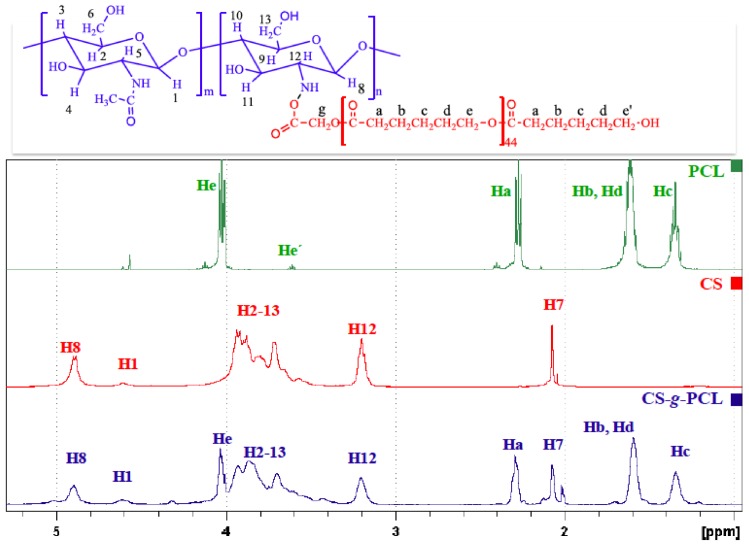
^1^H NMR CS, PCL and the synthesized graft copolymer CS-*g*-PCL.

The chemical structure of the CS-*g*-PCL copolymer was verified by both ATR-FTIR and ^1^H NMR spectroscopies. The ATR-FTR spectrum of the CS-*g*-PCL copolymer displays both the characteristic band of the ester group of PCL at 1723 cm^−1^ and the characteristic amide bands of CS at 1650 cm^−1^ and 1576 cm^−1^ (Figure 1) suggesting the successful grafting of the PCL chains along the CS backbone. The ^1^H NMR spectrum of the CS-*g*-PCL copolymer shows the methylene peaks of PCL at 4.03 (He), 2.30 (Ha), 1.59 (Hb, Hd) and 1.34 (Hc) ppm and the broad pyranose hydrogen peaks of chitosan between 3.7–3.9 ppm, verifying the presence of both components in the copolymer structure (Figure 2). ^1^H NMR spectroscopy was also employed to determine the degree of grafting of the PCL chains along the chitosan backbone by rationing the peak integrals of the Ha protons of PCL at 2.38 ppm to the pyranose protons H12 and H7 of chitosan at 3.2 and 2.07 ppm. ^1^H NMR analysis yields that 1 PCL chain is grafted per 111 CS repeat units, which correspond to 78 wt % CS in the graft copolymer.

### 3.2. Cell Morphology Visualized by Scanning Electron Microscopy 

**Figure 3:**
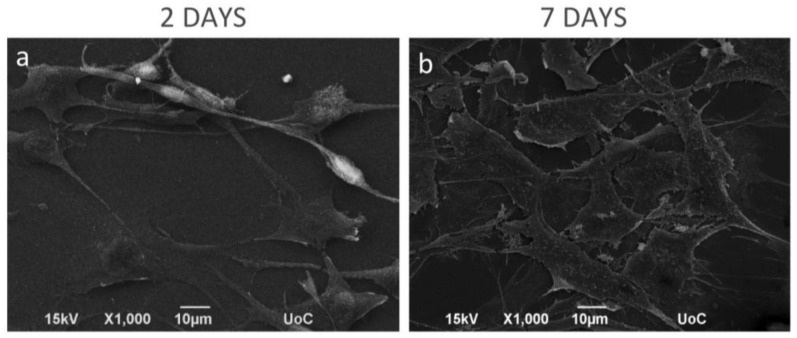
Scanning electron microscopy (SEM) images showing the morphology of MC3T3-E1 pre-osteoblastic cells on the CS-*g*-PCL surfaces, 2 and 7 days after seeding. SEM images illustrating MC3T3-E1 cell adhesion and growth on the copolymer surface: (**a**) After 2 days of seeding, a few cells were grown on the material surface, but they retain their elongated-fibroblastic morphology; (**b**) On day 7, cells have extensively proliferated forming a thick layer on the whole polymer surface. Original magnifications are 1000× and scale bars represent 10 μm.

The morphology of the MC3T3-E1 pre-osteoblastic cells on the CS-*g*-PCL surface was investigated by SEM on days 2 and 7 after cell seeding and is shown in Figure 3. On day 2 (Figure 3a) a few adherent cells on the material surface were observed, but they retain their characteristic osteoblastic morphology, interacting both with the substrate and with each other. Moreover, the cells appear elongated with a spindle-like shape. After 7 days in culture (Figure 3b), the pre-osteoblastic cells have extensively proliferated and expand to form a thick layer that covers the whole polymer surface. The cells appear flattened with wide lamellipodia formation and long cellular extensions of the cell membrane. This morphology is characteristic for pre-osteoblastic cells cultured on a preferable, biocompatible material surface. Τhe strong initial adhesion and the consequent proliferation indicate that the CS-*g*-PCL surfaces favor the growth of pre-osteoblastic cells. 

### 3.3. Confocal Microscopy

**Figure 4:**
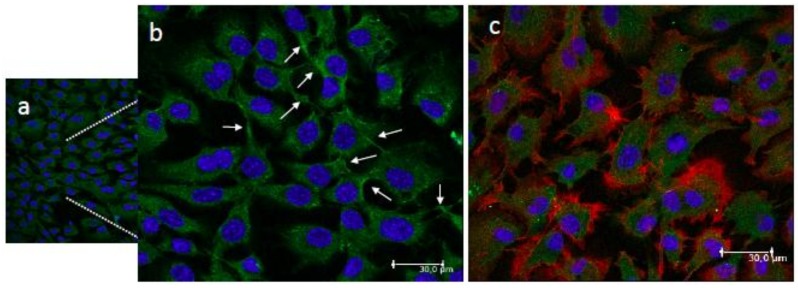
Confocal fluorescence microscopy images showing the adhesion and morphology of MC3T3-E1 pre-osteoblastic cells on the CS-*g*-PCL surfaces 3 days after seeding ((**a**,**b**); (**b**) is a magnified area of (**a**)). Cytoskeletal organization of cells visualized via actin (red), vinculin (green) and nucleus (blue) staining. White arrows show the vinculin focal adhesion points (**b**). An overlay of the three staining is depicted in image (**c**). Cells strongly attach on the material surface and form cell-cell interactions, covering the whole surface area. Original magnifications are 20× in (**a**) and 40× in (**b**) and (**c**) with scale bars representing 30 μm.

The morphology of the adhered MC3T3-E1 pre-osteoblastic cells cultured on the CS-*g*-PCL polymer surfaces for three days was examined using a confocal fluorescence microscope. The cytoskeletal organization of the cells was visualized via actin (red), vinculin (green) and nucleus (blue) staining (Figure 4). Figure 4b is a magnified area of Figure 4a, while Figure 4c depicts an overlay of the three stains. The cells attach strongly on the material surface and form cell-cell interactions. Moreover, they retain their elongated morphology with wide lamellipodia formation and long cellular extensions of the cell membrane. The observed fully spread cell morphology with spindle shape and cytoplasmic extensions signal a strong adhesion profile on the polymer surfaces. 

### 3.4. Cell Viability and Proliferation

**Figure 5:**
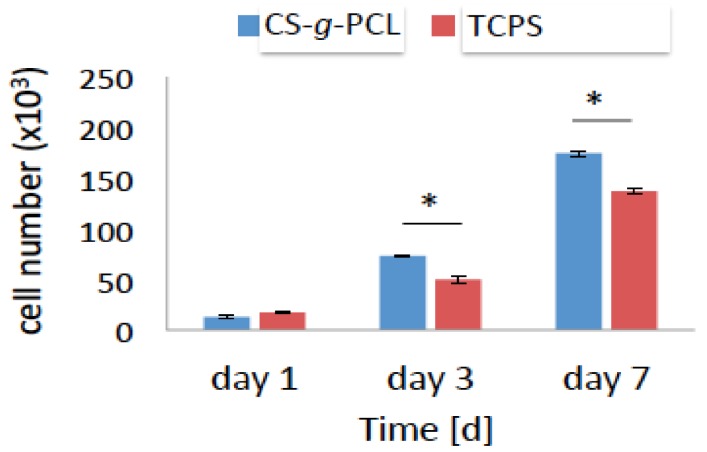
Viability and proliferation assay showing the number of the MC3T3-E1 pre-osteoblastic cells cultured on the CS-*g*-PCL surface and on tissue culture treated polystyrene (TCPS) after 1, 3, 7 days in culture. Optical density values of viability are normalized to the cell number using a calibration curve. The number of cells cultured on the CS-*g*-PCL surfaces was similar to those on TCPS 1 day after seeding but after 3 and 7 days it was significantly higher for the cells cultured on the CS-*g*-PCL copolymer surface.

The viability and proliferation of the MC3T3-E1 pre-osteoblastic cells on the CS-*g*-PCL substrates was quantitatively determined using the colorimetric PrestoBlue^®^ assay, 1, 3 and 7 days after cell seeding (Figure 5). On day 1, the number of viable cells was similar for the CS-*g*-PLC and TCPS surfaces and no significant differences were observed. However, viable cell numbers on the copolymer surface were significantly higher compared to TCPS after 3 and 7 days of culture (Figure 5). At each time point, cell viability was calculated following the normalization of the absorbance units to that obtained for the cells cultured on TCPS (control) and was expressed as a percentage to the control (100%) (Figure 5). On day 1, the viability of the cells cultured on the CS-*g*-PCL surfaces was 80% and increased to 146% and 127% after 3 and 7 days in culture, respectively. These results indicate that the CS-*g*-PCL surfaces promote the viability and proliferation of the pre-osteoblastic cells. 

### 3.5. Osteogenic Cell Response on the CS-g-PCL Surfaces 

#### 3.5.1. Early Markers of Osteogenesis

**Figure 6:**
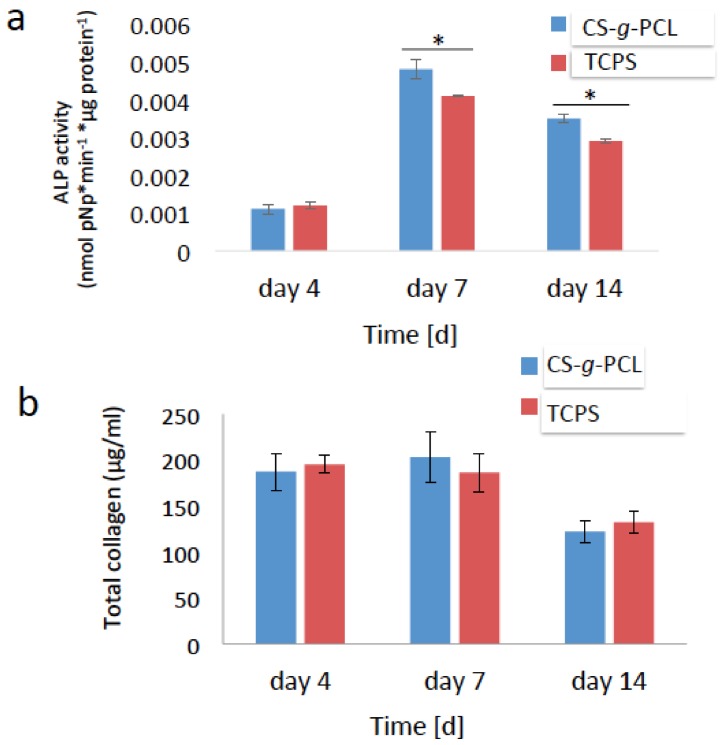
Early markers of osteogenesis: (**a**) Alkaline phosphatase (ALP) activity of the MC3T3-E1 cells cultured on the CS-*g*-PCL surface and TCPS for 4, 7 and 14 days. The ΑLP activity of the cells cultured on the CS-*g*-PCL was significantly increased compared to TCPS after 7 and 14 days; (**b**) Levels of collagen in the supernatants of the MC3T3-E1 cells cultured on the CS-*g*-PCL surface and on TCPS for 4, 7 and 14 days. The graft copolymer enhanced the production of collagen on day 7 compared to the control TCPS. Error bars represent the average of triplicates ± SEM of two independent experiments (*n* = 6).

In order to examine the potential of the MC3T3-E1 pre-osteoblasts cultured on the CS-*g*-PCL polymer surfaces to differentiate into mature osteoblasts in vitro, the alkaline phosphatase (ALP) activity and the secretion of collagen were measured as early markers of osteogenesis. For these experiments, the cells were cultured in the presence of osteogenic medium. The ALP activity of the cells cultured on the copolymer surface was normalized to the protein concentration and shown in Figure 6a. No significant differences were observed in the ALP activity of cells cultured for 4 days on CS-*g*-PCL substrates compared to TCPS surfaces. However, the ΑLP activity of the cells cultured on the CS-*g*-PCL copolymer was significantly higher compared to that of the cells cultured on TCPS, both after 7 and 14 days. In both cases, the ALP activity was higher on day 7 and decreased from day 7 to day 14. Moreover, the amount of collagen secreted from the cells into the supernatant was measured after 7 and 14 days of culture and is shown in Figure 6b. The graft copolymer enhanced the production of collagen on day 7 compared to the TCPS control and exhibited lower, but comparable to the control, collagen levels after 14 days of culture.

#### 3.5.2. Late Markers of Osteogenesis

**Figure 7:**
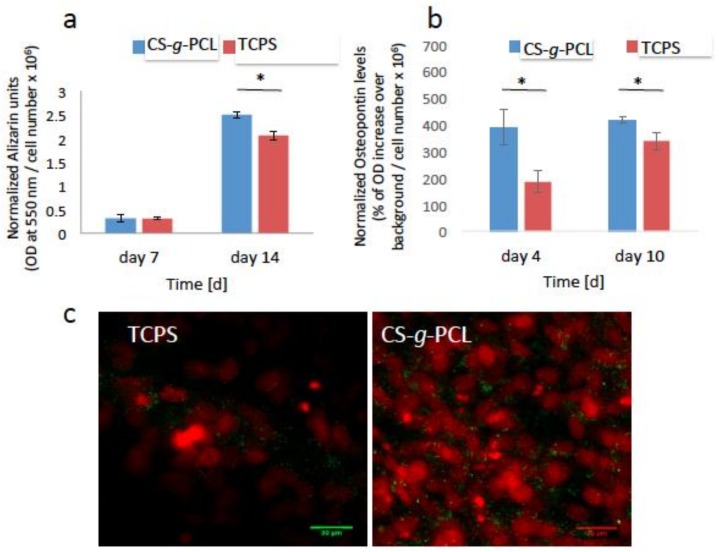
Late markers of osteogenesis normalized by the cell number: (**a**) Calcium biomineralization by Alizarin Red S staining of the MC3T3-E1 cells cultured for 7 and 14 days on CS-*g*-PCL and TCPS. The matrix mineralization of the copolymer substrate was significantly increased compared to TCPS after 14 days of culture; (**b**) Measurement of the endogenous levels of osteopontin of the pre-osteoblastic cells cultured on the two material surfaces for 4 and 10 days. Osteopontin levels were significantly higher on the CS-*g*-PCL surfaces compared to TCPS after 4 and 10 days in culture; (**c**) Confocal microscopy images demonstrate the endogenous expression of osteopontin on the two material surfaces 10 days after seeding. Osteopontin is depicted in green around the red cell nuclei. The expression is higher for cells cultured on the CS-*g*-PCL surface compared to TCPS 10 days after seeding. Original magnifications are 40× and scale bars represent 30 μm. Error bars represent the average of triplicates ± SEM of two independent experiments (*n* = 6).

Calcium deposits through mineralization is a specific marker of the late stages of cell differentiation. As described previously, Alizarin Red was used to stain the calcium deposits in the extracellular matrix of the pre-osteoblasts after 7 and 14 days of culture in osteogenic medium. In order to normalize the calcium deposits to the cell number, the number of living cells was measured using the PrestoBlue^®^ assay, prior to the Alizarin Red staining. Figure 7a shows that the extracted calcium-dye complex by CPC for the cells cultured on the CS-*g*-PCL surfaces was similar to that of TCPS after 7 days of culture. Moreover, the matrix mineralization for the copolymer surface was significantly increased compared to that for TCPS after 14 days of culture. 

Finally, the expression of endogenous osteopontin was detected as another marker of osteogenesis. As described previously, osteopontin is a phosphorylated glycoprotein involved in bone mineralization. In our study, the endogenous expression of osteopontin for the cells cultured on the CS-*g*-PCL and TCPS surfaces, for 4 and 10 days, was detected using in-cell enzyme-linked immunosorbent assay (ELISA) and confocal laser fluorescence microscopy (CLFM). As shown in Figure 7b, the levels of osteopontin were significantly higher on the copolymer surface in contrast to those cultured on TCPS after 4 and 10 days. This result was confirmed by the confocal microscopy images (Figure 7c) showing osteopontin in green color around the red cell nuclei. The expression of endogenous osteopontin is higher for the cells cultured on the CS-*g*-PCL surfaces compared to TCPS 10 days after seeding. 

## 4. Discussion

The development of biomaterials for bone tissue engineering aims to provide favorable substrates for osteoblastic and mesenchymal stem cells to facilitate their functions such as cellular viability, proliferation, migration and differentiation towards tissue regeneration [22,23]. Ceramics were widely used as bone implants due to their stability and mechanical strength, but their bioactivity and compatibility are under investigation [24,25]. On the other hand, many studies have indicated the enhancement of osteogenesis both in vitro and in vivo using biodegradable polymeric materials [26,27]. All these factors determine the material selection for the fabrication of the suitable scaffolds. Chitosan is a well-known natural polymer derived from chitin, the second most abundant polymer in nature. Chitosan has been extensively employed in tissue engineering applications due to its advantageous properties [7]. Many studies have shown that following blending or graft polymerization of chitosan with other polymers, the biological, mechanical and degradation characteristics are enhanced [28]. In this direction, PCL is a suitable synthetic polymer for blending with chitosan due to its low melting point and good tensile properties [11]. Young et al. have shown that CS-PCL blends can form membranes, which were used to fabricate a bioengineered corneal endothelium and therefore, facilitated the corneal endothelial cell (CEC) transplantation in vivo [29]. Moreover, Prasad et al. showed that the adhesion, viability and proliferation of human keratinocytes (HaCaT) and mouse fibroblasts (L-929) cultured on electrospun chitosan/PCL blends were enhanced and proposed the use of these blends as appropriate biomaterials for skin tissue engineering [11]. 

Previous studies from our group have focused on the synthesis of a CS-*g*-PCL copolymer, bearing PCL chains grafted along the chitosan backbone. This copolymer was used as a matrix for the development of Wharton’s jelly mesenchymal stem cells from three different donors and the results showed excellent cellular response [21]. In addition, evaluation of the immunomodulatory potential of CS-*g*-PCL copolymers with different compositions by investigating the differentiation of primary bone marrow derived macrophages (BMDM) showed that the materials promote the anti-inflammatory activity and the transition of M1 to M2 macrophages [30]. Interestingly, the observed anti-inflammatory effect increased with increasing chitosan content from 50 to 78%, a finding that makes the latter composition attractive for further investigations as the immunomodulation is crucial for the prediction of the fate of a developed biomaterial to be used as an implant. 

Based on our previous work, the aim of this study was to evaluate the in vitro biological response of MC3T3-E1 pre-osteoblastic cells on the CS-*g*-PCL surfaces. These cells, which can be easily obtained and cultured, have the capacity to differentiate into osteoblasts and osteocytes in vitro [31]. First, the adhesion of the cells on the CS-*g*-PCL surfaces after 2 and 7 days of culture was examined by SEM analysis (Figure 3). Cells reached confluence on day 7 and retained their characteristic fibroblastic phenotype with a highly elongated spindle-like shape. This result is in accordance with previous studies reporting the promoting of the adhesion of bone marrow mesenchymal stem cells, fibroblasts, keratinocytes and corneal endothelial cells on CS-PCL blends [5,11,28,32]. Initial cell adhesion is a critical step in tissue engineering because it mediates subsequent events such as cell viability and proliferation [33]. In our study, the viability and proliferation of pre-osteoblastic cells cultured on the copolymeric surfaces were significantly increased compared to TCPS after 3 and 7 days in culture (Figure 5). Similarly to our copolymeric material composition that supports the pre-osteoblastic proliferation increase, Young et al. showed a good growth of corneal endothelial cells reaching confluence on CS-PCL membranes with a composition of 75–25% demonstrating sufficient strength and suitability as a carrier in culture [29]. In another study, He et al. report that the growth of bone marrow mesenchymal stem cells cultured on CS-PCL membranes was better on the 30–70% composition compared to the 100% PCL, which could be attributed to amino groups on the CS-PCL composites which impart more hydrophilic sites than on the pure PCL, thus resulting in more suitable conditions for cell growth [5]. 

Our results indicate that the CS-*g*-PCL graft copolymer supports the osteogenic differentiation of the pre-osteoblastic cells. This was confirmed by a significant increase in the ALP activity of cells cultured on the CS-*g*-PCL surfaces compared to TCPS (Figure 6a), which is in line with the notion that chitosan promotes the osteogenic differentiation of cells as reported by He et al. who show that the ALP activity of bone marrow mesenchymal stem cells cultured on CS-PCL 30–70% membranes was higher compared to pure PCL [5]. Moreover, increased levels of extracellular collagen at early stages of culture were measured (Figure 6b), indicating a healthy extracellular matrix formation on the copolymeric surface, which is essential for tissue regeneration. Interestingly, Young et al. showed that bovine corneal endothelial cells cultured on a CS-PCL 75–25% membrane exhibited the highest production of collagen type IV, compared to the CS-PCL 85–15% and 90–10% blends [29]. Finally, the increased expression of osteopontin as well as the higher calcium deposits for the cells cultured on the CS-*g*-PCL surfaces indicate that the copolymeric material promotes the mineralized matrix formation. 

Previous studies have reported that the composition of chitosan and polycaprolactone in composites made from these two components is crucial as high concentrations of chitosan can favor the proliferation, the osteogenic differentiation potential and the anti-inflammatory activity [5,28,29,30]. We therefore focus in this study on the in vitro biological assessment of a CS-*g*-PCL copolymeric material containing 78% *w*/*w* chitosan, which was employed as two-dimensional substrates in an osteogenic cell model. Our data demonstrate that the CS-*g*-PCL copolymeric substrates are capable to promote the viability, proliferation and osteogenic differentiation of pre-osteoblastic cells in vitro. Since our next goal is the in vivo application of customized three-dimensional CS-*g*-PCL copolymeric scaffolds as attractive candidates in clinical translation, our efforts focus on the fabrication of scaffolds, which can elicit a strong osteogenic response in vivo, however, this is the subject of another work. Preliminary data of our group in this direction indicate promising results on the in vitro biological response of human bone marrow derived mesenchymal stem cells cultured on CS-*g*-PCL substrates. 

## 5. Conclusions

This study described the in vitro biological response of pre-osteoblastic cells on CS-*g*-PCL copolymer surfaces. Cells cultured on the material surface retain their characteristic fibroblastic morphology, interacting both with the substrate and with each other. The viability and proliferation of the cells cultured on the CS-*g*-PCL surfaces for 3 and 7 days was significantly increased compared to TCPS. Moreover, the increased levels of specific osteogenic markers of the cells cultured on the copolymer substrates indicate that the CS-*g*-PCL copolymer promotes the differentiation of pre-osteoblastic cells into mature osteoblasts, demonstrating the potential of the graft copolymer for scaffold fabrication in bone tissue engineering applications.
